# Rationale and design of the AlloFIST trial: a phase I/IIa study to evaluate dose escalation of allogeneic adipose-derived stroma/stem cells for the treatment of Crohn’s fistula

**DOI:** 10.1136/bmjopen-2025-104517

**Published:** 2025-12-29

**Authors:** Etienne Buscail, Cyrielle Gilletta de Saint Joseph, Marine Lebrin, Ines Frument, Fabian Gross, Barbara Bournet, Louis Buscail, Adrian Culetto, Fatima Mokrane, Marie Charlotte Delchier, Isabelle Quelven, Charline Daguzan, Grégory Pugnet, Jean Pierre Duffas, Laurent Ghouti, Antoine Philis, Nicolas Carrere, Benoit Lepage, Guillaume Le Cosquer

**Affiliations:** 1 Colorectal Surgery, University Hospital Centre Toulouse, Toulouse, France; 2 Digestive Health Research Institute, Toulouse, Occitanie, France; 3 Centre d’investigation clinique de Toulouse, Toulouse, Occitanie, France; 4 Gastroenterology, University Hospital Centre Toulouse, Toulouse, France; 5 Gastroenterology, CHU Toulouse, Toulouse, France; 6 Radiology, University Hospital Centre Toulouse, Toulouse, France; 7 Pharmacy, University Hospital Centre Toulouse, Toulouse, France; 8 DRCI, University Hospital Centre Toulouse, Toulouse, France; 9 CHU Toulouse, Toulouse, France; 10 University Hospital Centre Toulouse, Toulouse, France; 11 Epidemiology, University Hospital Centre Toulouse, Toulouse, France

**Keywords:** Colorectal surgery, Inflammatory bowel disease, Mesenchymal Stem Cells

## Abstract

**Introduction:**

Crohn’s disease (CD) is a chronic inflammatory disorder of the gastrointestinal tract distinguished by progressive bowel damage with a risk of structuring and penetrating complications. It is characterised by focal or segmental transmural inflammation that disrupts intestinal mucosal integrity and favours the development of abscesses and fistulas. Perianal fistula develops in 13%–39% of patients with CD. Their care is difficult but improves with medical and surgical treatment to preserve anal continence and avoid a maximum proctectomy. Combined treatment with seton placement and concomitant anti-TNF (infliximab, adalimumab) allows wound healing in 40%–70% of cases. The currently available treatments are not curative and fail to provide a long-term resolution. The injection of adipose stromal cells is currently being evaluated in clinical studies for repair-damaged tissues in various diseases (limb ischaemia, osteoarthritis, systemic sclerosis, etc). Immunoregulatory and anti-inflammatory properties of AdMSC (adipose-derived stroma/stem cells) are responsible for accelerating healing and represent an innovative approach for treating perianal fistulas associated with CD.

**Methods and analysis:**

This phase I/IIa study is designed to assess the treatment of complex perianal fistulas linked with CD after failure of conventional treatment by injection of AdMSC (CellReady) into the fistula. Two doses of associated AdMSC will be tested for a dose escalation (5×10^7^ and 10×10^7^ cells) and injected into the wall of the fistula. Those eligible for inclusion include patients with controlled luminal CD characterised by a Harvey-Bradshaw score below or equal to eight and diagnosed on clinical, endoscopic, histological and/or radiological criteria, a colonoscopy dating back less than 1 year without ulcer in the rectum and presence of complex chronic perianal fistula with a maximum of two internal ports and three external ports. All patients must have social security insurance or equivalent social protection. The aim of this study is to determine the optimal dose corresponding to maximum efficacy 6 months after injection of cells with a treatment-related adverse event rate of 20%.

**Ethics and dissemination:**

The EU CT number 2024-511821-75-00 was approved by the following Ethics Committee: CPP (committee for the protection of persons in French: comité de protection des personnes) Ouest 1 – Tours #2024UEMED-18 and ANSM (French Agency for the Safety of Health and Medicinal Products in French : Agence nationale de sécurité du médicament et des produits de santé) #2024-511821-75-00 (Sponsor number RC31/13/7030, protocol V2.1). The results will be disseminated through conventional scientific channels.

**Trial registration number:**

NCT06636032.

The results will be disseminated through conventional scientific channels.

**STRENGTHS AND LIMITATIONS OF THIS STUDY**

- This fast-track dose-escalation study seeks to provide evidence on the safety and tolerability of two doses of associated AdMSC which will be tested for a dose escalation (5×10^7^ and 10×10^7^ cells).
- The originality of our study lies first and foremost in the use of the production process.
- Due to its single-arm design, this study does not allow direct outcome comparisons with a respective control group.

## Introduction

 Perianal fistulas are at the forefront (42% to 72.4%) of morbid complications of Crohn’s disease (CD), affecting nearly one third of patients and complicating abscesses in 35%–48% of cases. The current treatment is based on a combination of drainage (proctologic and surgical) and biologic (anti-TNF) techniques, but the failure rate varies from 30% to 80%.^1^ The literature shows that injection of autologous or allogeneic AdMSCs, after appropriate surgical and medical preparation, represents a promising combination strategy for the management of resistant perianal fistulas.^2^ However, multiple prospects (and unknowns) exist for the treatment of anoperineal fistulas in CD by cell therapy. The current problem lies with standardising trials by selection of the study population, route of administration, type (autologous, allogeneic or FSV) and number of cells (1 to 12×10^7^), mode and number of injections, volume injected, associated surgical procedures, clinical and radiological assessment criteria and immuno-monitoring. The relative robustness of allogeneic AdMSC trials in terms of patient numbers and follow-up opens the door to the use of allogeneic stem cells in this indication and enables systematic, traceable preparation of cells from healthy donors.^3^

We propose to regulate the injection conditions, volume, number of cells and evaluation mode (clinical and MRI) in a phase I/IIa study using allogeneic cells whose expansion and certification process is well-mastered and standardised.

The originality of our study lies first and foremost in the use of the production process mastered by Cell-Easy, a pharmaceutical company, in premises adapted to the production of Advanced Therapy Medicinal Products (ATMPs) and well-characterised (CellReady drug). This production process is both shorter in time (14 days), therefore reducing the number of passages of these cells and more efficient in terms of the number of cells obtained and their quality than that used in other studies conducted in the same field.^4 5^ This reduction in cell amplification duly preserves the immunomodulatory properties of native cells as much as possible while limiting the drift associated with culture processes. This experimental drug (CellReady) has been approved as part of a cell therapy protocol for the healing of digital ulcers in scleroderma (ADUSE study MTIMSANAT-2023-07-0016_2019-003906-28). This protocol therefore targets patients with complex CD-related anoperineal fistula previously managed with combined treatment (drainage on setons+anti-TNFα or biotherapies) according to an identical protocol for all patients, failing to heal after 6 months but with controlled intra-luminal disease. The rationale for this study is based on the immunoregulatory, anti-inflammatory and trophic properties of AdMSCs which could enhance and accelerate healing of fistulous tracts.^6 7^ AdMSCs are injected into the wall of the fistula tract. We aimed to assess the safety and effectiveness of AdMSCs for treatment-refractory complex perianal fistulas in patients with CD.

## Objectives

### Primary objective

The primary objective of this study is to determine the optimal dose of cells to inject, making it possible to obtain a rate of grade ≥2 treatment-related adverse events (AEs) below the threshold of 20% with the best clinical effectiveness 6 months after injection.

### Secondary objective

The secondary objectives are to evaluate:

- Effectiveness and safety up to 6 months after injection of the cells.
- Efficacy up to 6 months post-injection of the cells on clinical, biological and radiological criteria:
  - Clinical criteria: discharge, general examination, specific quality of life, disease activity, fistula classification, anal incontinence, symptoms and impact of treatment on perineal lesions.
  - Biological criteria: blood count, inflammation, liver function, coagulation.
  - Radiological criteria: disappearance or persistence of the fistula tract, presence of fibrous sequelae, T2 hypersignal of the tract, enhancement following gadolinium injection into the tract(s) and presence or absence of abscesses.
- The therapeutic effect obtained depends on the quantity of AdMSC injected.

## Method and analysis

### Study design

This is a phase I/IIa, single-centre (Toulouse university hospital), open-label study using a cell therapy drug injected in situ into the fistula tract.

It is a dose-escalation study testing two different doses of AdMSC: (5×10^7^ and 10×10^7^ cells). Switching to the higher dose will take place after a meeting of the Independent Monitoring Committee (IMC) after 6 months of follow-up. Dose escalation is defined a priori from an ‘up-and-down’ scheme on 3 cohorts of 3 patients, determined using a ‘Bayesian Optimal Interval (BOIN) Design’ approach.^8^

The trial will be organised in line with the phase I methodology described by Storer^9^ and Zhou *et al*.^8^ The dose will be assessed 6 months after injection of the last patient in each group according to an a priori defined dose escalation scheme (up-and-down type). Escalation to the higher dose, maintenance at the same dose or de-escalation will occur after a meeting of the IMC for each cohort of 3 patients. Prior to each ISC meeting (for each patient group), the efficacy rate and number of AEs will be estimated using a continuous reassessment method.^10^

Previous studies by Korean and Spanish teams have evaluated the feasibility, safety and efficacy of the cells.^4 5^ Here, we are carrying out a phase I/II study aimed at determining the optimal dose, including both efficacy and safety criteria. The 6-month assessment allows an additional 4 months to highlight any AEs attributable to the treatment. However, it is not considered necessary to extend this period for the analysis of the primary endpoint, given the low risk of AEs expected after this 6-month follow-up period.

The final dose selected for a subsequent clinical phase will be the one that achieves the best efficacy with a serious AE rate under 20%.

This approach presents certain constraints since success and AE rates need to be recalculated regularly as patients are recruited. The dose escalation scheme is defined a priori. However, calculations of efficacy and AE rates can be executed at successive stages to provide objective quantitative elements for discussion by the IMC, which may or may not decide to continue with the dose escalation scheme as defined a priori.

### Dose escalation procedure—sample size calculation

The optimal dose will be determined 6 months after the injection of the last patient in the last group: after completing the full dose escalation schedule with 9 patients (3 patients per group), we will select the dose for which under 20% of grade ≥2 treatment-related AEs have been reported and for which healing is most complete. The probability of observing AEs at 6 months will be estimated using a continual reassessment method described by Seeger *et al*.^10^ Follow-up visits will take place 1, 3 and 6 months after cell injection.

A total of 9 patients will be included. Each patient will receive the pre-determined dose of stem cells after conventional treatment combining drainage with setons and biotherapy with infliximab or adalimumab. This treatment will be carried out identically for each patient.

The following up-and-down scheme will be applied. It has been defined using the ‘BOIN Suite’ application, available at https://www.trialdesign.org. The parameters used to define the scheme are as follows:

- Calculation based on a non-informative a priori distribution.
- Doses evaluated (dose h=50 million and dose h+1: 100 million).
- Starting at the lowest dose (h).
- 3 cohorts of 3 patients.
- Probability of target dose limiting toxicity (defined as the proportion of grade ≥2 AEs): 20%.

**Figure 1:**
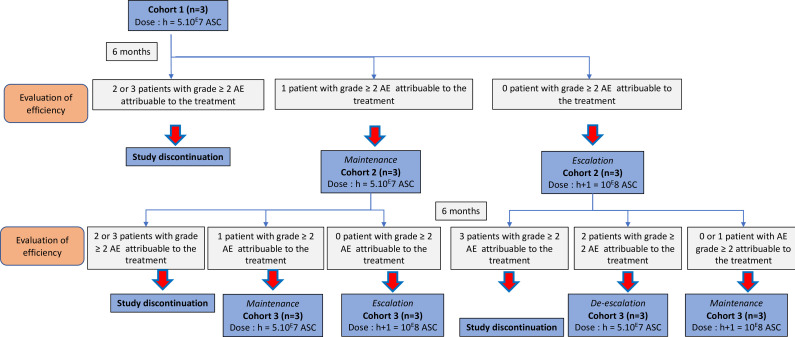
Study flow-chart.

The decision table (online supplemental table 1) associated with these parameters is as follows (from https://www.trialdesign.org/one-page-shell.html#BOIN):

The flow chart is detailed in the figure below (figure 1).

The study is continued until 9 patients are included, unless the following early stopping rules are applied:

- Cohort 1 (patients 1 to 3): discontinuation if two or 3 patients observed with an AE grade ≥2 related to treatment.
- Cohort 2 (patients 4 to 6): discontinuation if ≥3 patients with an AE grade ≥2 related to treatment are observed among all patients recruited.
- Cohort 3 (patients 7 to 9): discontinuation if ≥4 patients with an AE grade ≥2 related to treatment among all recruited patients are observed.

After 6 months of follow-up has been completed for the last patient in each group, the Independent Supervisory Board meets to decide whether to switch the next group to the higher dose, maintain the dose or de-escalate. Only safety criteria (number of patients with AE grade ≥2) can allow the dose to be increased.

### Experimental design

In this study, patients are followed up over six visits.

The pre-injection/injection period comprises two visits:

- Selection visit (V0).
- Inclusion visit (V1) 2 weeks maximum before injection.

The injection period comprises two visits:

- Visit 2 (V2) fistula preparation visit, including examination under anaesthesia, fistula curettage.
- Visit 3 (V3) injection of AdMSCs.

The follow-up period comprises 3 visits at 1 month, 3 months and 6 months post-injection (V4, V5, V6).

**Figure 2:**
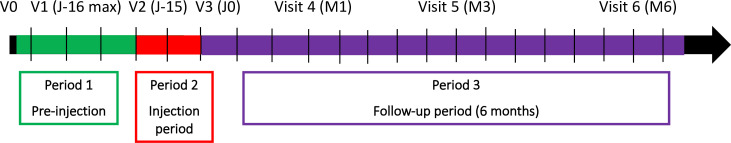
Study Timeline.

**Figure 3:**
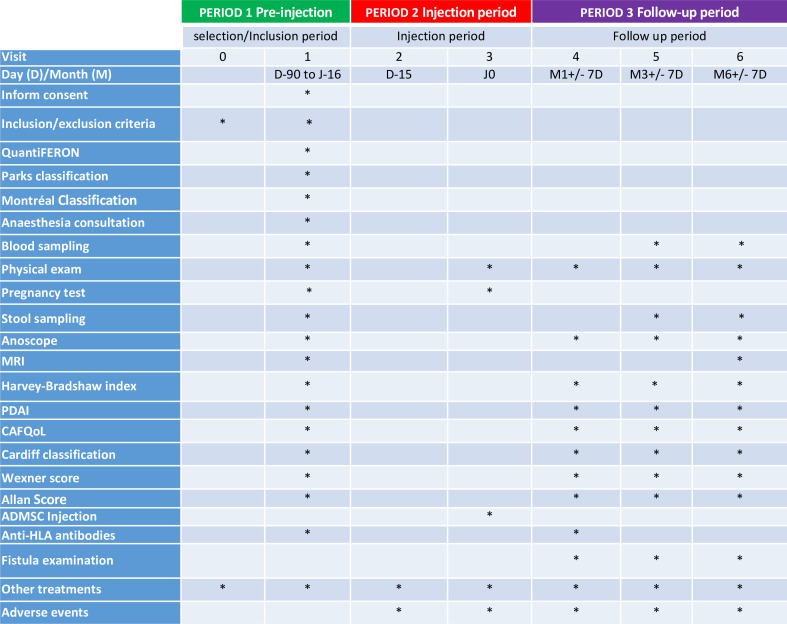
Study visit. PDAI, perineal disease activity index.

Study assessment and study time line are detailed in figure 2 and table figure 3.

### Study population

#### Inclusion criteria

- Patients must meet all the following criteria in order to be eligible to participate in the study.
- Patients over 18 years old.
- Patients who signed the informed consent.
- Patient affiliated to a social security system.
- Controlled luminal CD characterised by a Harvey-Bradshaw score below or equal to eight and diagnosed on clinical, endoscopic, histological and/or radiological criteria.
- Colonoscopy dating back less than 1 year without ulcer in the rectum.
- The fistula had to have a maximum of two internal and three external openings.
- Patient treated at least 6 months by advance therapy (anti TNF, anti-integrin, anti-interleukin 12/23, anti-interleukin 23, JAK inhibitors) and drainage with one or more setons, with good drainage confirmed by the absence of residual collection or an undrained tract on MRI.

#### Exclusion criteria

Any patient who meets the following criteria is not to be enrolled in this study:

- Refusal of the patient to participate in the study.
- Positive QuantiFERON test.
- Patient with transplanted organ.
- History of cancer in the last 5 years or lympho-proliferative disease.
- Persistent bacterial or viral infection.
- Patient with a contraindication to MRI.
- Known allergy to gadolinium.
- Known allergy to albumin.
- End-stage organ failure.
- Pregnant or breastfeeding women.
- Women of childbearing age without effective contraception.
- Patient under judicial protection, under guardianship or curatorship.

### Injection method (surgical procedure)

The selected patients undergo a preliminary examination under anaesthesia to prepare the fistula, with cleaning/curettage of the fistula tract(s). The purpose of this preliminary surgical phase is to confirm effective drainage of the suppuration and to eliminate granulous tissues from the fistula tract(s). The injection of the cells is performed during a second procedure under anaesthesia 2 weeks after the first. Patients are managed by outpatient hospitalisation sessions for both surgical procedures. The AdMSC are thawed and prepared in syringes on the day of injection. The transport conditions and quality of the product are checked in the pharmacy and then in the operating room. The doses will be stored in nitrogen gas at Cell-Easy (−150°C). A few days before a patient’s injection, the frozen cell doses will be shipped to the Toulouse hospital pharmacy specialised in ATMPs management in a special dry-shipper container containing liquid nitrogen with continuous temperature monitoring. The thawed cells will be stored at 2–8°C until they are dispensed to the investigator team for injection within a maximum of 10 hours, in accordance with the stability studies conducted on the reconstituted product in the syringe. Transport conditions will comply with Good Transport Practices (in accordance with European Directive 2004/23/EC). Frozen cell doses will be stored at Cell-Easy and then sent to the Toulouse hospital pharmacy specialised in ATMPs on the day of a patient’s injection. The thawed product in the syringe will be checked by a pharmacist and then dispensed by authorised personnel. All checks are carried out by the manufacturer before shipment and then by the hospital pharmacist on receipt and before dispensing. A single practitioner from the team (EB) performs all the injections to ensure that the technique is homogeneous and comparable.

The injection protocol is standardised in line with the ADMIRE CD study^3^: curettage/cleaning of the tract(s), removal of the seton(s) and then closure of the internal opening(s) with an ‘X’ stitch of absorbable suture or a mucosal or musculo-mucosal advancement flap if the internal opening appears large and/or fibrous.

Four or eight 2.5 mL vials (12.5×10^6^ AdMSC/vial) containing a total of 50 or 100 million AdMSC are then taken up into two 10 mL syringes. For 50 million cells, there are two 10 mL syringes, and for 100 million cells, there are four 10 mL syringes. The final volume of the cell suspension is 2.5 mL (overfilled to 2.7 mL) and the medium consists of DMEM containing human albumin and Cryostor CS 10. The content of two of the syringes is injected submucosally into the internal opening(s) and the remaining half is injected into the wall of all fistula tract(s). At the end of the procedure, the skin around the external openings is massaged to promote cell diffusion and the external openings are covered with a dry dressing.

At the end of the surgery, patients are hospitalised for 24 hours for monitoring. At the end of this period, if notable AEs have occurred, the patient is then allowed to go home with a simple prescription for analgesics and no specific requirements for local care.

### Evaluation

The patients are followed up during consultations at 1, 3 and 6 months. At the time of inclusion, Montreal phenotypic classification is used to identify the different presentations of CD and Parks classification is used to characterise fistula tracts.

The Cardiff classification is used at the time of inclusion and compared at 1-month, 3-month and 6-month follow-up visit to evaluate the evolution of the anoperineal disease. Anoperineal disease activity is determined with the perineal disease activity index (PDAI), anal continence is assessed by determining Jorge and Wexner score, luminal disease activity is identified using the Harvey–Bradshaw index and quality of life is evaluated with CAF-QoL score.

A control anoperineal MRI scan is performed at the inclusion and 6-month follow-up visits to ascertain the radiological response. MR studies will be evaluated by a single referring radiologist with 15 years of experience in pelvic disease and CD.

### Outcomes

#### Primary endpoint

The optimal dose will be determined by the following combined criteria at 6 months:

- No AEs of grade ≥2 attributable to the experimental treatment (CellReady).
- Efficacy of AdMSC treatment on fistula tract healing as defined by a complete clinical and radiological response. Clinical efficacy corresponds to absence of flow through the inner or external orifice observed on an anoscopy and through the external orifice(s) while radiological efficacy refers to the disappearance of fistula tract or fistula tract present but inactive (on pelvic MRI).

The clinical response will be defined as follows:

- Complete: non-catheterizable closure of all external openings without pressure relief.
- Partial: non-catheterizable closure of ≥ 50% of the external opening pathway without pressure relief from any of the openings.
- Failure: closure of < 50% of the external openings ± persistence of pressure relief from one or all of the external openings ± appearance of a new tract or abscess."

The radiological response will be defined as follows:

- Complete: fibrous sequelae with no T2 hypersignal or enhancement after the injection of gadolinium into the tract(s) and no abscesses.
- Partial: decrease of the inflammatory signal (T2 hypersignal and enhancement after the injection of gadolinium) ± the length ± the largest diameter of the tracts, with no abscesses.
- Failure: absence of radiological modification or occurrence of a new fistula tract±abscesses

#### Secondary endpoints

The efficacy and adverse effects are assessed at 1 month, 3 months and 6 months post-injection.

Safety is determined through the collection of AEs (related or not) occurring throughout the trial.

The effectiveness criteria will also be evaluated:

- At 1, 3 and 6 months on clinical and biological criteria.
- And at 6 months on radiological criteria (MRI).

Changes will be ascertained between the inclusion visit and follow-up visits.

##### Clinical criteria

- Treatment effectiveness measured by the absence of flow through the internal orifice visualised during an anoscopy and through the external orifice(s) at V1, V4 (M1), V5 (M3), V5 (M6).
- Evolution of quality of life measured by CAFQoL between V1 and V4 (M1), V5 (M3), V6 (M6). This questionnaire measures quality of life specifically for patients with anal fistula in CD.
- Evolution of the Harvey-Bradshaw disease activity score (abdominal examination) between V1 and V4 (M1), V5 (M3) and V6 (M6).
- Evolution of the fistula on clinical examination and search for abnormal tissue formation at V1 and V4 (M1), V5 (M3) and V6 (M6).
- Evolution of the PDAI between V1 and V4 (M1), V5 (M3) and V6 (M6).
- Evolution of the Cardiff classification between V1 and V4 (M1), V5 (M3) and V6 (M6).
- Evolution of the Wexner anal incontinence score between V1 and V4 (M1), V5 (M3) and V6 (M6).
- Evolution of the Allan Score (patient symptoms and impact of treatment on perineal lesions) between V1 and V4 (M1), V5 (M3) and V6 (M6).

##### Radiological criteria (pelvic MRI)

- Efficacy of treatment on MRI: disappearance of fistulous tracts or persistent but inactive fistulous tract between V1 and V6 (M6).

##### Biological criteria

- Evolution of biological parameters: CBC, Platelets, CRP, hepatic enzyme profile, PT, APTT, albumin between V1 and V5 (M3) and V6 (M6).

##### Description of characteristics at inclusion

Characterisation of anal fistulas using the following two classifications:

- Evaluation of the Parks classification at inclusion.
- Evaluation of the Montreal classification at inclusion.

### Sample size calculation

In this type I/IIa dose-finding study, we aimed to assess two doses (5×10^7^ and 10×10^7^). We plan to include a minimum of 3 patients (in case of early stop at the first dose because of safety) to a maximum of 9 patients, as described in figure 1. Dose escalation, maintenance or de-escalation was defined applying a ‘Bayesian Optimal Interval (BOIN) Design’ for 3 cohorts of 3 patients, with a target toxicity probability of 0.20.^8^

### Data analysis

We will describe the number of included patients, potential loss to follow-up and dose escalation using a flow chart. The rate of grade ≥2 AEs attributable to the treatment and the proportion of complete clinical and radiological efficacy will be estimated using a Bayesian continuous reassessment method^10^ by applying a non-informative prior. Details regarding the clinical and radiological response will be described (complete/partial response and failures). The statistical distribution of the secondary criteria will be described for each follow-up visit. AEs will be described using the MedDRA classification by AdMSC dose according to their causality and severity. The study is scheduled to begin in May 2025 and end in November 2026.

### Ethics and safety

This study will be conducted according to the principles of Good Clinical Practice or the ethical principles stated in the most recent version of the Declaration of Helsinki.

The study was approved by the French Ethics Committee (Comité de Protection des Personnes CPP Ouest 1 – Tours #2024UEMED-18) and European Medicines Agency with the EudraCT number 2024-511821-75-00. The study is registered on the website clinicalTrials.gov under the number NCT06636032. The informed consent document (online supplemental file 2) with study information will be used to explain to patients in simple terms what participation in the study means for them. All patients will be asked to sign a written informed consent form received by the principal investigator.

### Patient and public involvement

Patients and/or the public were not involved in the development of this study except for the informed consent validated by a patient association.

## Data collection/management

### Clinical data

All the information required by the protocol must be entered in an electronic data capture system. The investigator will ensure the accuracy, completeness and timeliness of the reported data. A complete audit trail on all data changes will be maintained. The investigator or designee will cooperate with the monitor and the data manager for the periodic review of data in order to ensure the accuracy and completeness of the electronic data capture system at each scheduled monitoring visit and before any submission of results. The data are collected on an electronic case report form. All the information will be contained in the original documents, or in the authenticated copies of said documents; and relating to clinical examinations, observations or other activities conducted as part of a research study and necessary for the reconstitution and evaluation of the research. The documents in which the source data are saved are called the source documents.

## Quality control

A clinical researcher appointed by the Sponsor will regularly visit each investigating centre during the implementation of the research one or several times during the research phase according to the frequency of the inclusions and at the end of the research phase. During these visits and in line with the risk-based monitoring plan (participant, logistics, impact, resources), the following will be reviewed:

- Informed consent.
- Compliance with the research protocol and the procedures defined therein.
- Quality of the data collected in the electronic data capture system: accuracy, missing data, consistency of the data with source documents (medical records, appointment books, originals of laboratory results and so on).
- Management of the ATMP.

All visits will be the subject of a written monitoring report.

## Ethics and dissemination

Study information will be publicly available at www.clinicaltrials.gov (NCT06636032). The results of this trial will be submitted for publication in relevant peer-reviewed publications and the key findings presented at national and international conferences. Dissemination will be done under the responsibility of the study’s Coordinating Investigator with the agreement of the Principal Investigators. The co-authors of the report and publications will be the investigators and clinicians involved on a pro rata basis of their contribution to the study as well as the statistician and methodologist.

Sharing of data generated by this project is an essential part of our proposed activities and will be carried out in several different ways. We would wish to make our results available to the community of scientists interested in colorectal disease to avoid unintentional duplication of research and improve the current standard of care. The EU CT number 2024-511821-75-00 was approved by the following Ethics Committee: CPP (committee for the protection of persons in French: comité de protection des personnes) Ouest 1 – Tours #2024UEMED-18 and ANSM (French Agency for the Safety of Health and Medicinal Products in French: Agence nationale de sécurité du médicament et des produits de santé) #2024-511821-75-00 (Sponsor number RC31/13/7030, protocol V2.1).

## Patient and public involvement

## Supplementary material

10.1136/bmjopen-2025-104517 online supplemental file 1

10.1136/bmjopen-2025-104517 online supplemental file 2

## References

[1] Schwartz DA, Loftus EV, Tremaine WJ (2002). The natural history of fistulizing Crohn’s disease in Olmsted County, Minnesota. *Gastroenterology*.

[2] Buscail E, Le Cosquer G, Gross F (2021). Adipose-Derived Stem Cells in the Treatment of Perianal Fistulas in Crohn’s Disease: Rationale, Clinical Results and Perspectives. *Int J Mol Sci*.

[3] Panés J, García-Olmo D, Van Assche G (2016). Expanded allogeneic adipose-derived mesenchymal stem cells (Cx601) for complex perianal fistulas in Crohn’s disease: a phase 3 randomised, double-blind controlled trial. Lancet.

[4] Guadalajara H, Herreros D, De-La-Quintana P (2012). Long-term follow-up of patients undergoing adipose-derived adult stem cell administration to treat complex perianal fistulas. Int J Colorectal Dis.

[5] Lee WY, Park KJ, Cho YB (2013). Autologous adipose tissue-derived stem cells treatment demonstrated favorable and sustainable therapeutic effect for Crohn’s fistula. Stem Cells.

[6] Grégoire C, Lechanteur C, Briquet A (2017). Review article: mesenchymal stromal cell therapy for inflammatory bowel diseases. *Aliment Pharmacol Ther*.

[7] Bourin P, Bunnell BA, Casteilla L (2013). Stromal cells from the adipose tissue-derived stromal vascular fraction and culture expanded adipose tissue-derived stromal/stem cells: a joint statement of the International Federation for Adipose Therapeutics and Science (IFATS) and the International Society for Cellular Therapy (ISCT). Cytotherapy.

[8] Zhou Y, Li R, Yan F (2021). A comparative study of Bayesian optimal interval (BOIN) design with interval 3+3 (i3+3) design for phase I oncology dose-finding trials. Stat Biopharm Res.

[9] Storer BE (2001). An evaluation of phase I clinical trial designs in the continuous dose-response setting. Stat Med.

[10] Seegers V, Chevret S, Resche-Rigon M (2011). Dose-finding design driven by efficacy in onco-hematology phase I/II trials. Stat Med.

