# Supplementary material for: Rationale and design of the AlloFIST trial: a phase I/IIa study to evaluate dose escalation of allogeneic adipose-derived stroma/stem cells for the treatment of Crohn’s fistula
Source: BMJ Open. 2025 Dec 29;15(12):e104517. doi: 10.1136/bmjopen-2025-104517 (PMC12750760; doi:10.1136/bmjopen-2025-104517)
Supplement: online supplemental file 1 [file bmjopen-15-12-s001.docx]

| **Escalation/De-escalation Rule** | | | | | | | | | | |
| --- | --- | --- | --- | --- | --- | --- | --- | --- | --- | --- |
| **Number of evaluable patients treated** | **1** | **2** | **3** | **4** | **5** | **6** | **7** | **8** | **9** |  |
| Escalate if # of grade ≥ 2 TRAEs is greater or equal to… | 0 | 0 | 0 | 0 | 0 | 0 | 1 | 1 | 1 |  |
| De-escalate if # of grade ≥ 2 TRAEs is greater or equal to… | 1 | 1 | 1 | 1 | 2 | 2 | 2 | 2 | 3 |  |
| Eliminate if # of grade ≥ 2 TRAEs is greater or equal to… | NA | NA | 2 | 3 | 3 | 3 | 4 | 4 | 4 |  |

**Table 1:** Escalation/De-escalation Rule; TRAEs: Treatment-Related Adverse Events.
