# Supplementary material for: Rationale and design of the AlloFIST trial: a phase I/IIa study to evaluate dose escalation of allogeneic adipose-derived stroma/stem cells for the treatment of Crohn’s fistula
Source: BMJ Open. 2025 Dec 29;15(12):e104517. doi: 10.1136/bmjopen-2025-104517 (PMC12750760; doi:10.1136/bmjopen-2025-104517)
Supplement: online supplemental file 2 [file bmjopen-15-12-s002.pdf]

Document d'information à l'attention du participant à la recherche

ETUDE DE PHASE I/II DE THERAPIE CELLULAIRE EN ESCALADE DE DOSE DANS LA PRISE EN CHARGE DES FISTULES ANO-PERINEALES LIEES A LA MALADIE DE CROHN

Etude AlloFIST

CODE PROMOTEUR : RC31/13/7030

Promoteur de la recherche :	Investigateur coordonnateur :
**CHU de Toulouse**<br>Hôtel Dieu, 2, rue Viguerie TSA 80035, 31059 TOULOUSE cedex 9<br>Tel : +33 (0)5 61 77 86 03	**Dr Etienne BUSCAIL**<br>Service de Chirurgie digestive, Pôle Digestif, Hôpital Rangueil, 1 Avenue Jean Poulhès,<br>TSA 50032, 31059 Toulouse Cedex 9<br>Tél : + 33 (0)5 61 32 23 73<br>Courriel :buscail.e@chu-toulouse.fr

PARTIE 1 : INFORMATIONS SUR LA RECHERCHE

* *Les mots ou groupes de mots surmontés d'un astérisque sont inclus dans le glossaire*

Madame, Monsieur,

L'investigateur vous propose de participer à un essai clinique* qui a pour objectif d'évaluer la sécurité de l'injection dans les fistules ano-périnéales de la maladie de Crohn, des cellules souches issues du tissu adipeux d'un donneur (tissu graisseux abdominal) appelées AdMSC. Cet essai a également comme objectif de déterminer la dose permettant d'obtenir la meilleure efficacité de fermeture des fistules 6 mois après leur injection.

Vous pouvez prendre le temps nécessaire pour lire les informations ci-dessous, discuter avec vos proches et votre médecin traitant et poser toutes vos questions au médecin de la recherche, appelé investigateur*. Après avoir obtenu les réponses satisfaisantes à vos questions et disposé d'un délai suffisant de réflexion, vous pourrez alors décider si vous acceptez de participer à la recherche ou non. Si vous acceptez de participer à cette recherche, vous devez compléter et signer le formulaire de consentement de participation. Un exemplaire du document complet vous sera remis.
Votre participation est entièrement volontaire. Si vous ne désirez pas prendre part à cet essai clinique vous continuerez à bénéficier de la meilleure prise en charge médicale possible, conformément aux connaissances actuelles.

Pourquoi cette recherche est-elle mise en place ?

Cette étude vous est proposée dans le cadre de la prise en charge de votre maladie de Crohn. Il s'agit d'une maladie inflammatoire chronique de l'intestin dont la cause reste inconnue à ce jour et qui évolue par poussées successives entrecoupées de rémissions. Lorsque l'activité inflammatoire n'est pas bien contrôlée, la maladie de Crohn peut notamment provoquer des fistules dans la zone ano-périnéales. Il s'agit de lésions, sous forme de tunnels, qui relient le rectum ou l'anus à la peau. Les fistules sont généralement traitées de manière chirurgicale par drainage des abcès puis assèchement, avec comblement des fistules jusqu'à l'obtention de leur fermeture, et par un traitement médical qui contrôle l'inflammation.

Cependant malgré un traitement approprié et bien conduit, il est fréquent que ces fistules persistent. Cet échec thérapeutique peut s'expliquer par la complexité du drainage chirurgical, de leur infection et/ou par la difficulté du contrôle médicamenteux de l'inflammation. Chez les patients atteints comme vous de la maladie de Crohn, en échec des traitements classiques des fistules ano-périnéales, de nouvelles approches thérapeutiques, complémentaires au traitement médical usuel, peuvent être utilisées : il s'agit de la thérapie cellulaire.

Les cellules stromales mésenchymateuses issues du tissu graisseux/adipeux (AdMSC) ont déjà montré un effet bénéfique sur la cicatrisation des fistules dans la maladie de Crohn. En effet, un médicament contenant ce type de cellules est autorisé dans cette indication et est utilisé en soin courant : il s'agit de l'Alofisel® (Darvadstrocel). Ce médicament est actuellement retiré du marché de l'Union européenne. Ces cellules sécrètent des molécules capables d'agir sur l'inflammation, le système immunitaire et la formation de vaisseaux sanguins. Pour les vertus qu'elles semblent pouvoir apporter, cette population de cellules issues du tissu adipeux va être utilisée dans l'étude qui vous est proposée.

En complément du traitement médical dont vous bénéficiez, nous vous proposons aujourd'hui de participer à cette étude de thérapie cellulaire conduite au sein du Centre Hospitalier Universitaire de Toulouse. Tous les patients inclus dans l'étude bénéficieront du traitement de thérapie cellulaire. L'injection de ces cellules se fera directement dans la paroi des fistules au bloc opératoire.

<u>En quoi la recherche consiste-t-elle ?</u>

L'objectif principal de cette recherche est de déterminer la dose optimale de cellules à injecter. La détermination de cette dose optimale est basée à la fois sur des paramètres de sécurité évalués à partir des évènements indésirables recueillies et sur la meilleure efficacité clinique de fermeture des fistules, 6 mois après l'injection du médicament expérimental chez des patients atteints de la maladie de Crohn en échec des traitements classiques.

Les objectifs secondaires de cette étude sont d'évaluer l'efficacité et les effets indésirables du traitement à 1 mois, 3 mois et 6 mois après l'injection des cellules et d'évaluer la quantité d'AdMSC injectée par rapport à l'effet thérapeutique obtenu.

9 patients seront inclus dans cette étude qui se déroulera dans le centre hospitalier universitaire (CHU) de Toulouse. La durée de participation pour chaque participant est de 6 mois.

Cette étude est financée par la Direction Générale de l'Offres de Soins (DGOS) via un Programme Hospitalier de Recherche Clinique interrégional (PHRCi).

Quel est le traitement étudié ?

Le traitement proposé dans cette étude réside en une administration dans le trajet fistuleux d'AdMSC allogéniques (c'est-à-dire de cellules issues du tissu graisseux/adipeux d'un donneur). La dose administrée sera de 50 millions ou de 100 millions de cellules.

Cette escalade de dose sera effectuée sur trois groupes successifs de trois patients. Chaque patient au sein d'un même groupe bénéficiera de la même dose de cellules.

Le premier groupe de 3 patients recevra la dose de 50 millions de cellules. Après 6 mois de suivi du dernier patient du groupe 1, un comité de surveillance indépendant du protocole (CSI), se réuni afin de décider du passage au groupe suivant (groupe 2) à la dose supérieure de 100 millions de cellules ou du maintien à la dose de 50 millions de cellules.

Après 6 mois de suivi du dernier patient du groupe 2, le CSI se réunira afin de décider du passage au groupe 3 à la dose supérieure, au maintien de la dose, ou bien à la dose inférieure.

Le passage à la dose supérieure est basé sur un critère de sécurité : la survenu des événements indésirables ((EI) graves attribués au traitement à l'étude **(nombre de patient ayant eu un EI** de grade ≥ 2 **imputable au traitement)**.

Le schéma d'escalade de dose correspond au graphique ci-dessous :

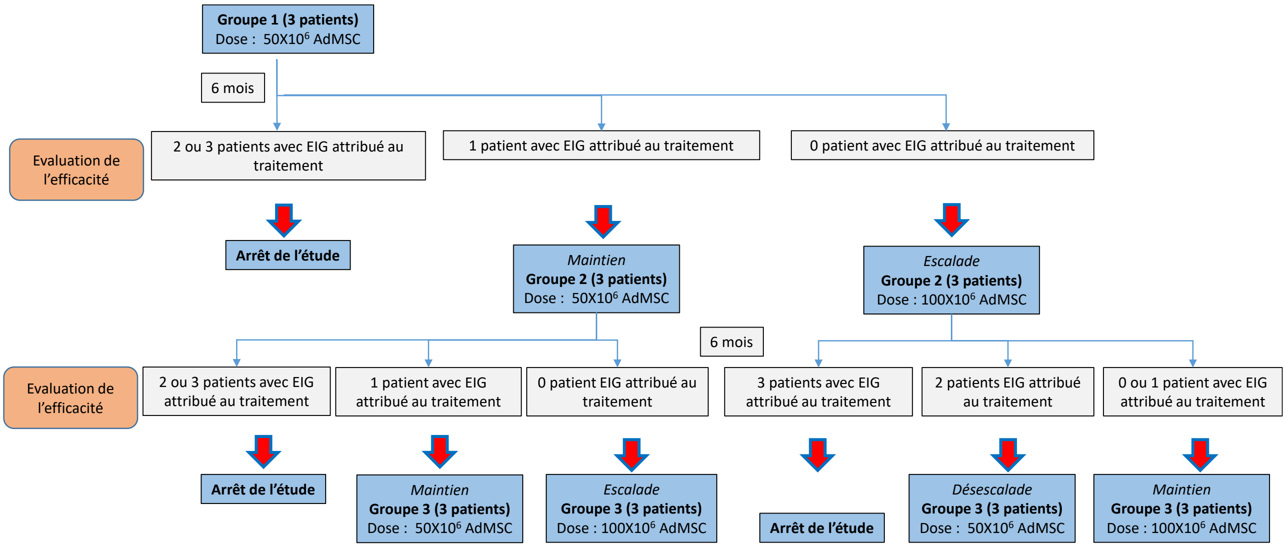

Il n'y aura pas de tirage au sort, tous les patients bénéficieront du traitement à l'étude. Outre le traitement par thérapie cellulaire étudié dans cette étude, vous poursuivrez les traitements médicaux qui vous auront été prescrits en fonction des recommandations en vigueur pour la prise en charge de votre maladie de Crohn. Certains traitements qui vous seront précisés par le médecin de l'étude seront en revanche interdits pendant l'étude (notamment ceux agissant sur le système immunitaire ainsi que les traitements à base de corticoïdes).

Qui peut participer ?

Pour participer à cette étude, vous devez être majeurs avoir signé un consentement éclairé et avoir un diagnostic confirmé de la maladie de Crohn luminale contrôlée et être pris en charge par un traitement combiné (drainage sur séton et anti-TNF) conventionnel après 6 mois. De plus, vous devez présenter une ou plusieurs fistules dites « complexes », avec un maximum de deux orifices internes et trois orifices externes. Vous devrez également avoir bénéficié d'une coloscopie de moins d'un an ne retrouvant pas d'ulcère dans le rectum. Vous devrez également être affilié(e) à un régime de sécurité sociale, ne pas présenter de contre-indication à l'IRM ou d'allergie connue au gadolinium ou à l'albumine, et ne pas avoir d'antécédent de cancer de moins de 5 ans ou d'infection persistante.

Si vous êtes une femme en âge de procréer, vous devez avoir un moyen de contraception efficace. Vous ne pourrez pas participer à cette étude si vous êtes enceinte ou si vous allaitez.

Pour recevoir le traitement à l'étude, il faudra que vous procédiez à certains examens et prélèvements sanguins complémentaires et que vos résultats soient compatibles avec la recherche (examen clinique, anuscopie, IRM…). Ces examens préalables auront lieu dans les services du pôle digestif de votre hôpital et le médecin de l'étude discutera avec vous des résultats.

Si vous participez à cette recherche, vous ne pourrez pas participer simultanément à une autre étude clinique et ce jusqu'à la fin de votre suivi dans l'étude soit 6 mois après les injections de cellules.

Votre participation à cette recherche biomédicale n'engendrera pour vous aucun frais supplémentaire par rapport à ceux que vous auriez dans le cadre du suivi habituel de cette maladie. Tous les frais liés à cette recherche sont pris en charge par le promoteur (frais liés à vos déplacements pour la visite d'injection). La participation à l'étude ne donne lieu par ailleurs à aucune indemnisation financière.

Comment la recherche se déroule-t-elle ?

Vous bénéficierez d'un examen médical avant le démarrage de la recherche qui déterminera si vous pouvez ou non y participer. Avant toute procédure reliée à l'étude, le médecin de l'étude vous expliquera le déroulement de la recherche et vous pourrez poser des questions. Vous devrez lire et signer le formulaire de consentement éclairé pour confirmer votre souhait de participer à l'essai clinique. Votre participation à la recherche durera 6 mois et nécessitera 6 visites à l'hôpital.

L’étude comporte 3 périodes :

- La période pré-injection :
    - Sélection (Visite 0) / Inclusion (Visite 1) : sélection puis inclusion dans l’étude (dès signature du formulaire de consentement éclairé).
- La période d’injection :
    - Visite 2 : préparation et nettoyage de la fistule sous anesthésie générale en ambulatoire.
    - Injection (Visite 3) : administration des AdMSC à l’hôpital Rangueil (Toulouse) lors d’une hospitalisation de 24h.

- La période de suivi : dure 6 mois après l’administration du traitement à l'étude et comporte 3 visites (les visites 4 à 6) réalisées à 1 mois, 3 mois et 6 mois après l’administration des cellules.

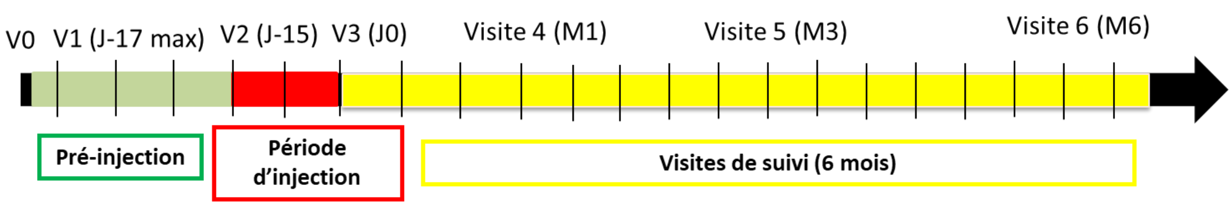

PERIODE 1 (visites 0, 1 et 2):

<u>Visite 0 :</u>

Vous serez sélectionné par le médecin en charge de l’étude au cours d’une consultation ou hospitalisation réalisée en soin courant. Après avoir lu cette notice d’information, vous disposerez d’un délai de réflexion avant de signer le consentement de participation à cette recherche.

<u>Visite 1 :</u>

La visite 1 d’inclusion se déroulera en hospitalisation de jour de votre centre de soins habituel (maximum 1 jour). Vous aurez au préalable bénéficié d’une visite de sélection au cours de laquelle le médecin vous aura présenté l’étude.

• Vous aurez ensuite un examen médical adapté à la recherche comprenant un relevé de vos antécédents médicaux et chirurgicaux, un examen clinique (mesures des pressions artérielles, fréquence cardiaque, température, poids, taille, fréquence respiratoire, examen des fistules), des examens complémentaires à visée gastroentérologique classique (IRM pelvienne, anuscopie) afin de valider votre inclusion dans l’étude.

Deux méthodes d’examen vont permettre de dresser une sorte de "carte routière" de la ou des fistules :

- L’IRM (Imagerie par Résonance Magnétique) pelvienne, technique d’imagerie sans rayons X, indolore et inoffensive. La durée de l’examen est de 30 à 45 minutes environ, durant lesquelles

vous devrez rester allongé dans la machine Cet examen peut toutefois être désagréable, notamment pour la sensation d'enfermement et le bruit que cet examen provoque.

- L'anuscopie, réalisée à l'aide d'un anuscope que l'on insère dans l'anus. Il permet l'examen visuel direct de la muqueuse du canal anal et du bas rectum. Cet examen, même s'il peut être désagréable, est sans douleur et ne nécessite aucune préparation particulière.

Une prise de sang sera effectuée pour les analyses suivantes :

- Pour vérifier le fonctionnement de votre foie, nous examinerons les enzymes hépatiques, l'albumine et la protéine C-réactive (mesure du niveau d'inflammation) (1tube de 5mL)
- Nous évaluerons également les facteurs de coagulation sanguine (1 tube de 2,7 mL),
- Comptage des composants du sang (Numération formule sanguine) et plaquettes pour évaluer votre système immunitaire (1tube de 3mL),
- Test de la tuberculose (QuantiFERON)(4 tubes de 1mL)
- Un dosage des Beta HCG sera réalisé pour les femmes en âge de procréer (1 tube de 2,5 mL).
- Dosage dans le sang de composés impliqués dans la maladie de Crohn (dosage des métabolistes du tryptophane) **:** 1 tube de sang de 7 mL sera prélevé.

Les échantillons seront pseudonymisés et conservés à -80°C pendant toute la durée de l'étude puis envoyés pour l'analyse à l'Equipe « Microbiote, Intestin et Inflammation » - UMRS 938 Centre de Recherche de Saint-Antoine, Faculté de Médecine - 27 rue de Chaligny 75012 Paris – France, sous la responsabilité du Pr Harry SOKOL. Les échantillons seront détruits après analyse.

- Recherche d'anticorps dirigé contre les cellules du donneur (anticorps anti-HLA) afin de s'assurer de l'absence de réaction immunitaire de votre organisme vis-à-vis des cellules du donneur (1 tube de 3,5mL).

Vos selles (3 mL) seront prélevées afin de doser une protéine (la calprotectine) et d'y étudier votre microbiote intestinal (micro-organismes présents dans votre intestin).

- **Etude du microbiote dans les selles:** Lors des visites 1 (inclusion), 5 (M3) et 6 (M6) vous apporterez un pot stérile contenant vos selles. Ces échantillons seront conservés dans des tubes contenant de la gélose et seront envoyés immédiatement pour analyse à l'Institut de Recherche en Santé Digestive (IRSD) (105 Av. de Casselardit, 31300 Toulouse) sous la responsabilité du Pr Nathalie Vergnolle

Vous devrez remplir trois questionnaires : un qui évalue votre qualité de vie (**CAF-QoL)**, un qui mesure l'activité de la maladie (**Index Harvey-Bradshaw**) et l'activité de votre maladie périanale **(PDAI).** Ces questionnaires pourront être remplis en 30 minutes environ.

Le médecin qui vous suit procédera à des interrogatoires afin d'établir des classifications et des scores de sévérité de votre maladie

A l'issue de ces examens et en fonction des résultats, le médecin investigateur confirmera ou non que vous pouvez participer à l'étude et programmera avec vous une nouvelle hospitalisation pour la visite 2.

Vous bénéficierez d'une consultation auprès d'un anesthésiste en vue de la visite d'injection réalisée sous anesthésie générale dans un délai de 60 jours à 16 jours avant la visite 3.

Période 2 : injection des cellules

Visite 2 : Préparation de la fistule

Un examen préliminaire sera réalisé en ambulatoire et sous anesthésie générale pour préparer et nettoyer la fistule 15 jours avant l'injection des cellules.

Visite 3 : Injection

Lors de la visite 3, vous serez hospitalisé(e) pour une durée de 24 heures pour l'injection des AdMSC allogéniques dans le trajet de votre fistule anale. Un dosage des Beta HCG sera réalisé la veille de l'intervention pour les femmes en âge de procréer (1 tube de 2,5 mL).
Un examen clinique complet sera à nouveau réalisé pour s'assurer que l'injection est possible. Cette injection sera réalisée sous anesthésie générale lors d'une hospitalisation d'au moins 24h. A l'issue de l'intervention chirurgicale vous serez admis(e) en salle de réveil puis dans le service de chirurgie digestive où vous bénéficierez de la surveillance post-opératoire habituelle du service avec suivi des signes infectieux.

Vous pourrez regagner votre domicile dès que votre état clinique le permettra.

PERIODE 3 (visites 4, 5 et 6):

Par la suite, vous devrez revenir régulièrement en consultation dans les services du pôle digestif 1, 3 et 6 mois (visites 4, 5 et 6) après l'injection des AdMSC. Il s'agit de la surveillance régulière classique des patients atteints de la maladie de Crohn et souffrant d'une ou plusieurs fistules anales.

Une évaluation clinique, une anuscopie, une évaluation de votre qualité de vie ainsi que les questionnaires permettant de mesurer l'activité de la maladie et la sévérité de la fistule traitée seront réalisés à chaque visite.
L'état de fermeture de votre fistule sera évalué à chaque visite.

Les examens et bilan suivants seront réalisés au cours de consultations (maximum une journée par visite):

- Anuscopie (visites 4, 5 et 6)
- Score et qualité de vie (visites 4, 5 et 6) : Index d'activité Harvey-Bradshaw, PDAI, questionnaire de qualité de vie (CAFQoL), classification de cardiff, score de Wexner, Score d'Allan)

- Recherche d'anticorps αHLA du donneur (afin de s'assurer de l'absence de réaction immunitaire de votre organisme vis-à-vis des cellules du donneur) (1 tube de 3,5mL)
- Recueil de selles : dosage de la calprotectine et étude de votre microbiote intestinal (bactéries présentes dans votre intestin) (visites 5 et 6),
- Bilan biologique détaillé plus haut (1tube de 5mL, 1 tube de 2,7 mL, et 1 tube de 3mL).
- IRM pelvienne (visite 6) 6 mois après l'injection des cellules.
- Dosage dans le sang de composés impliqués dans la maladie de Crohn (visites 5 et 6) (1tube de 7mL)

Lors de chaque visite, les traitements médicamenteux que vous prenez et les événements survenus au cours de l'étude seront relevés.

Participer à cette étude requiert votre adhésion et le respect du calendrier. Il est important que vous en discutiez avec l'investigateur (ou le médecin qui le représente) avant de décider de votre participation.

Que vous demandera-t-on ?

Le calendrier des visites avec les différents examens qui seront réalisés sont résumés dans le tableau ci-dessous :

	**PERIODE 1**		**PERIODE 2**		**PERIODE 3**		
	**Période de sélection/Inclusion**		**Période d'injection**		**Période de suivi**		
**Visites**	**0**	**1**	2	3	**4**	**5**	**6**
**Semaines**		**J-90 à J-16**	**J-15**	**J0**	**M1+/- 7j**	**M3+/- 7j**	**M6+/- 7j**
**Information de la recherche**	*						
**Consentement éclairé**		*					
**Antécédents médicaux/chirurgicaux**	*						
**Test tuberculose (QuantiFERON)**		*					
**Classifications de votre maladie et des fistules(Parks et Montréal)**		*					
**Consultation anesthésie**		*					
**Examens biologique**		*				*	*
**Examen clinique**		*		*	*	*	*
**Test de grossesse sanguin (si nécessaire)**				*			
**Recueil des selles**		*				*	*
**Anuscopie**		*			*	*	*
**IRM pelvienne**		*					*
**Indexs d'activité de la maladie (PDAI et Harvey-Bradshaw)**		*			*	*	*

**Qualité de vie (CAFQoL) et scores (Wexner et Allan)**		*			*	*	*
**Classification de Cardiff**		*			*	*	*
**Consultation anesthésie**		*					
**Nettoyage de la fistule**			*				
**Injection**				*			
**Anticorps αHLA du donneur**		*			*		
**Dosage de composés impliqués dans la maladie de Crohn**		*				*	*
**Evaluation fermeture de la fistule**					*	*	*
**Traitements concomitants**	*	*	*	*	*	*	*
**Evénements indésirables**			*	*	*	*	*

Si vous participez à cette recherche, vous devrez suivre les instructions suivantes :

- Déclarer au médecin de l'étude si vous avez participé à une autre recherche durant l'année précédente ou si vous faites actuellement partie d'une autre recherche. Ne pas prendre part à une autre recherche durant votre participation à cette recherche. Ces mesures ont pour but de vous protéger d'éventuels risques tels que des prélèvements sanguins trop nombreux, des interactions médicamenteuses, des expositions aux rayonnements ou d'autres dangers.
- Avoir en permanence sur vous la carte patient qui vous a été remise et que vous devrez présenter si vous êtes pris en charge par un autre médecin (en cas d'urgences ou de consultations chez un spécialiste par exemple).
- Si vous êtes traité par un autre médecin, il est important que vous indiquiez à l'équipe soignante votre traitement et le motif des consultations.
- Venir aux consultations prévues dans le cadre de la recherche.
- Signaler au médecin de l'étude, immédiatement ou lors d'une visite, tout évènement/effet indésirable même si vous pensez qu'il n'a pas de rapport avec la recherche en cours.
- Signaler au médecin de l'étude ou à l'équipe de recherche tous les traitements que vous prenez.
- Vous devez suivre les instructions du médecin sur tout traitement médicamenteux pris tout au long de l'essai (veuillez demander au médecin de l'étude pour plus de détails concernant les médicaments autorisés).
- Vous devez contribuer aux évaluations de l'étude aux visites spécifiques comme décrit dans ce document.

À tout moment, vous avez le droit de ne plus vouloir participer à cette recherche et de retirer votre consentement. Cette décision n'entraînera aucun préjudice et n'aura aucune conséquence sur votre prise en charge médicale à venir. Il est important que vous en informiez le médecin de l'étude. L'étude pourra être interrompue à tout moment sur décision du médecin en charge de l'étude, du Promoteur ou des Autorités de Santé. Les situations qui pourraient conduire à un arrêt de votre participation à la recherche sont présentées ci-dessous :

- Vous pouvez décider d'arrêter votre participation à la recherche en retirant votre consentement. Dans ce cas, les évaluations finales seront réalisées dès que possible. Toutes vos données et échantillons collectés avant votre retrait de consentement, y compris ceux collectés lors de l'évaluation finale, seront intégrés dans les analyses et les résultats de la recherche à des fins scientifiques. Il vous sera demandé de réaliser un suivi médical avec votre médecin traitant pour votre sécurité pendant au moins 3 mois pour s'assurer de l'absence d'effets indésirables en lien avec votre participation à cette recherche.
- Le médecin de l'étude peut décider de votre sortie anticipée de la recherche sans votre accord préalable s'il juge qu'il en est de votre intérêt : si votre état de santé s'aggrave ou ne s'améliore pas et qu'un traitement alternatif est médicalement indiqué, si le traitement à l'étude ou les procédures sont jugés dangereux (signe de « toxicité »), si vous n'êtes pas en mesure de participer correctement à la recherche comme indiqué, si le promoteur ou les autorités de santé décident de mettre fin prématurément à la recherche, pour toute raison non prévue qui rendrait nécessaire votre arrêt de participation à la recherche. Dans chacun de ces cas, le médecin de l'étude vous en expliquera les raisons.

En cas d'arrêt de la recherche une surveillance classique et optimale sera réalisée.

Quelles sont les éventuelles alternatives médicales ?

En cas de non-participation à la recherche, à la fin de la recherche ou en cas d'arrêt prématuré de la recherche, vous reprendrez votre suivi médical habituel par votre gastroentérologue qui décidera du traitement médical optimal à adopter pour votre pathologie (corticoïdes, traitements modulateurs de l'immunité, biothérapies).

Quels sont les bénéfices attendus et risques liés à la recherche ?

Il est possible que vous ne tiriez aucun bénéfice de votre participation à cette recherche. Cependant, il a déjà été démontré que ces cellules permettaient d'améliorer la cicatrisation des fistules anopérinéales. Vous pourrez également contribuer à apporter de nouvelles informations concernant le traitement des fistules anales de la maladie de Crohn dont d'autres patients ou la communauté scientifique et médicale pourraient bénéficier.

A titre global, les résultats de cette étude pourraient conduire à terme à envisager un nouveau traitement des fistules anales de la maladie de Crohn.

Le fait d'accepter de participer à cette étude implique d'accepter de venir en hospitalisation de jour ou en consultation pour les visites d'inclusion et de suivies à 1, 3 et 6 mois et en hospitalisation au moins 24h pour la visite d'injection. Le suivi proctologique est proche du suivi de tous les patients atteints de la maladie de Crohn et porteur de fistules anales. En plus du suivi classique, cette étude implique la réalisation d'une IRM et d'une opération réalisée sous anesthésie générale pour l'injection des cellules. Tous les frais supplémentaires liés à la recherche seront pris en charge par le promoteur de l'étude à savoir le CHU de Toulouse.

Les principaux inconvénients et risques que nous avons identifiés sont l'administration des AdMSC, les prises de sang, et la participation aux examens d'imagerie cérébrale :

- Prise de sang : vous pourrez ressentir un léger inconfort ou une douleur, et il existe un petit risque d'ecchymose et/ou d'infection au point de piqûre. Si vous avez déjà eu ce type de réactions, faites-le savoir au médecin de l'étude ou à l'infirmière.
- Examen d'imagerie IRM : il s'agit d'un examen indolore et sans danger. Lors de l'IRM pelvienne, vous pourriez trouver cet examen inconfortable si vous êtes claustrophobe (peur des espaces confinés). La plupart des gens trouvent cela surmontable, avec le soutien des techniciens de radiologie. Les risques liés à l'injection du produit de contraste (gadolinium) pendant l'IRM sont une hypersensibilité et une réaction allergique.
- Anuscopie : cet examen est généralement sûr, mais dans de rares cas, des complications mineures peuvent survenir (saignement mineur, légère déchirure péri-anale).
- L'administration des AdMSC :

Au cours de la réalisation des injections de cellules dans le trajet fistuleux, il peut y avoir là aussi des risques de rougeurs, de gonflement, d'hématomes ou de saignement local ainsi que la survenue d'une infection locale. Tous ces risques sont minimisés par l'expérience des chirurgiens et la surveillance particulière dont vous ferez l'objet. Votre hospitalisation dans la structure médico-chirurgicale permettra une surveillance adaptée tout au long de votre prise en charge. En cas d'arrêt de la recherche, une surveillance classique et optimale sera réalisée.

Les risques liés à l'anesthésie générale réalisée pour l'injection des cellules sont ceux d'une anesthésie générale classique: nausées et vomissements, maux de gorge ou enrouement passager provoqués par l'introduction d'un tube dans la trachée ou dans la gorge, traumatismes dentaires, rougeur douloureuse au niveau de la veine dans laquelle les produits ont été injectés, troubles passagers de la mémoire ou baisse des facultés de concentration dans les heures suivant l'anesthésie, complications imprévisibles. Ces risques sont minimisés par l'expérience des anesthésistes et la surveillance particulière dont vous ferez l'objet. Si vous acceptez de participer à l'étude, vous aurez une consultation spécifique avec l'anesthésiste et vous devrez signer un formulaire de consentement éclairé spécifique à la procédure d'anesthésie générale lors de la première visite.

Les études comportant ce type d'injection n'ont relaté aucun effet indésirable notable lié à l'injection de ces cellules. Il peut cependant survenir suite à l'injection des cellules : une élévation de la température corporelle (>37,5°C), des frissons/tremblements, des démangeaisons (prurit), une rougeur locale ou un gonflement local.

Vous devez informer le médecin de l'étude ou un membre de l'équipe soignante immédiatement si vous ressentez des effets secondaires. Si à tout moment, vous pensez présenter une réaction allergique, contactez immédiatement le médecin de l'étude. Si vous présentez des difficultés pour respirer, appeler le 15.

Vous ne pourrez pas participer à une autre recherche pendant toute la durée de l'étude.

Remboursement des frais

Les frais liés à la recherche sont entièrement pris en charge par le promoteur. La participation à l'étude n'est pas rémunérée.

Que se passera-t-il en cas d'arrêt prématuré de la recherche et après la recherche ?

A la fin de l'étude, soit 6 mois après les injections , vous suivrez une évaluation médicale adéquate complète prévue dans le protocole (examen clinique, évaluation de la fermeture de la fistule, questionnaire de qualité de vie, scores et classifications, bilan biologique, imagerie (IRM), anuscopie). Vous serez ensuite pris en charge dans la filière de soins courants conventionnelle.

Si vous devez sortir de l'étude prématurément (de votre décision ou de celle de l'investigateur), vous bénéficierez du même suivi que les patients ayant suivi l'étude de façon complète.

Vous pouvez décider d'arrêter à n'importe quel moment et quel qu'en soit le motif. Il est très important que vous preniez contact avec votre médecin pour l'informer de votre décision. Vous pourrez ainsi discuter ensemble des modalités d'arrêt du traitement et de la meilleure stratégie thérapeutique à adopter par la suite.

Votre médecin peut également décider à tout moment d'interrompre votre participation à la recherche s'il juge que cela est dans votre intérêt ; si vous ne suivez pas les recommandations de la recherche ou si la recherche est arrêtée. Les données et échantillons collectés avant cette interruption continueront à être utilisés pour la recherche.

Résultats Globaux de l'étude

A l'issue de l'étude et après analyse de toutes les données relatives à l'étude pour tous les patients, vous pourrez, si vous le demandez, être informé(e) des résultats globaux par l'intermédiaire du médecin qui vous suit dans le cadre de cet essai clinique conformément à l'article L.1122-1 et suivants du Code de la Santé Publique.

Une description en anglais de cette étude sera disponible dans le répertoire international des essais cliniques sur le site http://www.ClinicalTrials.gov.Vous pourrez accéder à ce site à tout moment. Les informations publiées sur ce site ne comportent aucun élément permettant de vous identifier.

Dispositions législatives et réglementaires

Conformément au règlement (UE) n°536/2014 du Parlement européen et du Conseil du 16 avril 2014 relatif aux essais cliniques de médicaments à usage humain :

- cette recherche a obtenu un avis favorable du Comité de Protection des Personnes le 18/07/2024 et l'autorisation de l'Agence Nationale de Sécurité du Médicament et des produits de santé (ANSM) le 12/09/2024,
- cette recherche entre dans le cadre de la Méthodologie de Référence MR-001 de la Commission Nationale Informatique et Libertés (CNIL)
- le Promoteur de cette recherche, le CHU de Toulouse, a souscrit une police d'assurance de responsabilité civile auprès de la société LLOYD'S INSURANCE COMPANY S.A. (Bastion Tower, Marsveldplein 5, 1050 Bruxelles, Belgique_ contrat HSLCET24004)
- le Promoteur assume l'indemnisation des conséquences dommageables de la recherche impliquant la personne humaine.
-

Lorsque la responsabilité du promoteur n'est pas engagée, les participants peuvent être indemnisés auprès de l'ONIAM (Office National d'Indemnisation des Accidents Médicaux, 36, avenue du général de Gaulle, 93175 Bagnolet cedex).

PARTIE 2 : INFORMATIONS SUR LES DROITS DU PARTICIPANT ET SUR LA GESTION DES DONNEES RECUEILLIES

Que signifie le principe d'un consentement libre et éclairé à la participation à un essai clinique ?

Votre participation à un essai clinique est libre et volontaire : vous êtes libre d'accepter ou de refuser de participer à cette recherche et vous pouvez interrompre à tout moment votre participation sans avoir à donner de raison et sans encourir aucune responsabilité ni préjudice de ce fait. Il vous suffit de le signaler à l'investigateur.

Votre décision de participer ou de ne pas participer n'aura aucune conséquence sur votre prise en charge médicale et la qualité de vos soins ou sur votre relation avec l'investigateur.

Pour participer à une recherche, vous devez donner préalablement votre consentement libre et éclairé. « Eclairé » signifie que vous aurez bénéficié d'une information claire et compréhensible sur les enjeux et le déroulement de la recherche et sur vos droits en tant que participant.

Vous serez informé(e) par l'investigateur qui vous suit de toute nouvelle information concernant la recherche qui pourrait modifier votre décision d'y participer.

Vous avez le droit d'obtenir communication, au cours ou à l'issue de la recherche, des informations concernant votre santé, détenues par l'investigateur ou, le cas échéant, le médecin ou la personne qualifiée qui le représente.

Durant la recherche, en cas de nécessité pour la continuité de vos soins, l'investigateur vous demandera de consentir au partage des informations strictement nécessaires avec votre médecin traitant.

Comment vos données personnelles* seront-elles traitées dans le cadre de la recherche ?
Si vous acceptez de participer à la recherche, vos données personnelles, y compris vos données de santé, feront l'objet d'un traitement* par le promoteur, en qualité de responsable du traitement de ces données.
Les données suivantes seront recueillies : données cliniques et biologiques, antécédents médicaux, données issues des échantillon biologiques, données issues de l'IRM et de l'anuscopie.
Conformément au Règlement Général de la Protection des Données (RGPD)*, le responsable du traitement des données*, le CHU de Toulouse, sur la base légale de l'exécution d'une mission d'intérêt public, peut effectuer un traitement* informatique de vos données personnelles*, de santé et de vos échantillons biologiques. Ce traitement est autorisé car il est nécessaire à des fins de recherche scientifique. Le responsable de traitement doit mettre en œuvre des mesures appropriées permettant de garantir vos droits et libertés, notamment le seul recueil de données strictement nécessaires à la recherche.

Les responsables du traitement (CHU de TOULOUSE) s'assurent notamment que les traitements de vos données soient réalisés conformément au Règlement (UE) 2016/679 Général sur la Protection des Données (RGPD) et aux dispositions de la loi relative à l'informatique, aux fichiers et aux libertés.

Seules les données strictement nécessaires aux objectifs de la recherche qui vous ont été présentés, seront recueillies et analysées.

Ces données seront conservées jusqu'à deux ans après la dernière publication des résultats de la recherche ou, en cas d'absence de publication, jusqu'à la signature du rapport final de la recherche. (cf MR001) puis seront archivées pendant 25 ans après la fin de l'étude conformément à la réglementation en vigueur.

Comment la confidentialité de vos données sera-t-elle assurée ?
Vos données personnelles seront traitées de manière confidentielle, conformément à la loi du 6 janvier 1978 modifiée dite « Loi Informatique et Libertés », et conformément au Règlement Général sur la Protection des Données (RGPD*).

Vos données seront codées*, c'est-à-dire que vous serez identifié par un numéro de code pour les besoins de la recherche, sans mention de vos noms et prénoms. Seul l'investigateur conservera la liste de correspondance entre le code et votre nom.

Qui aura accès à vos données dans le cadre de la recherche ?
Les informations concernant votre identité (nom, prénom) ne seront connues que par l'équipe médicale vous prenant en charge ainsi que par les personnes réalisant le contrôle de la qualité de la recherche mandatées par le promoteur, par les autorités sanitaires ou de contrôle, par le délégué à la protection des données du promoteur si vous le contactez à l'adresse dpo@chu-toulouse.fr et, en cas de litige, par le personnel habilité de l'organisme d'assurance du promoteur.
Ces personnes sont soumises au secret professionnel.

Vos données codées seront accessibles aux personnes suivantes :
- Le promoteur et les personnes agissant pour son compte,

- L'Institut de Recherche en Santé Digestive (IRSD)
- Centre de Recherche de Saint-Antoine (UMRS 938)
- Les experts indépendants chargés de ré-analyser les données pour vérifier les résultats de la recherche, en vue de leur publication, dans des conditions strictes de sécurité.

Ces personnes, soumises au secret professionnel, auront accès à vos données codées dans le cadre de leur fonction et en conformité avec la réglementation.

<u>Quels sont vos droits relatifs à vos données personnelles ?</u>

Conformément aux dispositions de la Loi relative à l'Informatique, aux fichiers et aux Libertés (loi n° 78-17 du 6 janvier 1978 modifiée par la loi n° 2018-493 du 20 juin 2018) et au Règlement Général sur la Protection des Données (règlement UE 2016/679), vous avez un droit d'accès et de rectification de vos données. Vous pouvez également demander la limitation du traitement de vos données (c'est-à-dire demander au promoteur de geler temporairement l'utilisation de vos données).

Même si vous acceptez de participer à la recherche, vous pourrez à tout moment vous opposer au traitement de vos données aux fins de réalisation de la recherche. Dans ce cas, aucune information supplémentaire vous concernant ne sera collectée.

Vous pouvez également exercer votre droit à l'effacement sur les données déjà recueillies mais celles-ci pourront ne pas être effacées si cela rendait impossible ou compromettait gravement la réalisation des objectifs de la recherche.

De plus, certaines données visant à assurant la qualité et la sécurité de la recherche (par exemple : les effets indésirables des produits testés) doivent obligatoirement être collectées par le promoteur. Vous ne pourrez pas exercer votre droit d'opposition ou d'effacement concernant ces données.

Vous pouvez également accéder directement ou par l'intermédiaire d'un médecin de votre choix à l'ensemble de vos données médicales en application des dispositions de l'article L. 1111-7 du Code de la Santé Publique. Tous ces droits s'exercent par écrit auprès du médecin qui vous suit dans le cadre de la recherche et qui connaît votre identité.

<u>Comment exercer vos droits ?</u>

Vous pouvez exercer vos droits à tout moment et sans avoir à vous justifier.

Le promoteur n'ayant pas accès à votre identité, il est recommandé de vous adresser, dans un premier temps, à l'investigateur, aux coordonnées disponibles dans la présente note.

Vous pouvez en outre, si vous le souhaitez, exercer vos droits auprès du délégué à la protection des données du promoteur par l'adresse dpo@chu-toulouse.fr qui gèrera cette demande en coordination avec le médecin et les professionnels impliqués dans l'étude. Dans ce cas, votre identité (prénom, nom) sera rendue accessible au délégué à la protection des données du promoteur.

Dans l'hypothèse où vous ne parvenez pas à exercer vos droits, vous disposez également du droit de déposer une réclamation concernant le traitement de vos données personnelles auprès de la Commission nationale de l'informatique et des libertés (CNIL), qui est l'autorité de contrôle compétente en France en matière de protection des données.

Vos données codées et vos données associées aux échantillons biologiques pourront-elles être réutilisées ?

Vous pouvez accepter ou refuser le principe de l'utilisation de vos données codées lors de recherches ultérieures, conduites exclusivement à des fins scientifiques dans le domaine des maladies digestives.

Avant que vos données ne soient réutilisées, vous recevrez une nouvelle note d'information stipulant les objectifs de chaque nouvelle étude nécessitant leur usage et le type de données qui seront utilisées afin que vous puissiez vous y opposer comme l'impose le Règlement Général de la Protection des Données (article 14). Cette/ces recherche(s) ultérieure(s) devra(ont) soit être conforme(s) à un référentiel établi par la CNIL si elle(s) entre(nt) dans le cadre d'une procédure simplifiée du fait de ses/leurs caractéristiques, soit faire l'objet d'une autorisation de la CNIL.

Toute l'équipe vous remercie et se tient à votre disposition pour répondre à vos questions.

GLOSSAIRE

**Essai clinique**	Un essai clinique est une recherche portant sur des médicaments à usage humain (Règlement Européen N°536/2014)
**Promoteur**	Personne physique ou morale responsable de la recherche, qui en assure la gestion et qui vérifie que son financement est prévu.
**Investigateur**	Personne physique chargée de surveiller et de diriger la recherche sur un lieu de recherche.
**RGPD**	Règlement Général sur la Protection des Données.<br>Règlement (UE) 2016/679 du Parlement européen et du Conseil du 27 avril 2016 relatif à la protection des personnes physiques à l'égard du traitement des données à caractère personnel et à la libre circulation de ces données
**Résultats globaux**	Résultats de la recherche résultant de l'analyse de toutes les données de celle-ci.
**Données personnelles**	Donnée se rapportant à une personne physique identifiée ou identifiable. Les données de santé sont des données à caractère personnel particulières car sensibles.
**Données codées**<br>**Ou pseudonymisées**	Le codage ou la pseudonymisation consiste à remplacer les données directement identifiantes (nom, prénom, etc.) d'un jeu de données par des données indirectement identifiantes (alias, numéro, etc.).
**Méthodologie de référence (MR)**	Procédure simplifiée encadrant l'accès aux données de santé pour les promoteurs de recherche
**Traitement des données**	Un traitement de données personnelles est une opération, ou ensemble d'opérations, portant sur des données personnelles, quel que soit le procédé utilisé (collecte, enregistrement, organisation, conservation, adaptation, modification, extraction, consultation, utilisation, communication par transmission ou diffusion ou toute autre forme de mise à disposition, rapprochement).
**Responsable du traitement**	C'est la personne morale (le CHU de Toulouse) qui détermine les finalités et les moyens d'un traitement, c'est à dire l'objectif et la façon de le réaliser.

FORMULAIRE DE CONSENTEMENT

ETUDE DE PHASE I/II DE THERAPIE CELLULAIRE EN ESCALADE DE DOSE DANS LA PRISE EN CHARGE DES FISTULES ANO-PERINEALES LIEES A LA MALADIE DE CROHN

Etude Allofist

Je soussigné(e) …………………………………………………………………………………………….(nom, prénom) consens librement à participer à cette recherche telle que décrite dans la lettre d'information et je confirme les points suivants :

- J'ai eu le temps de lire ces informations, de réfléchir à l'étude et j'ai obtenu des réponses appropriées à mes questions.
- J'ai bien été informé de la nature des objectifs de la recherche, des risques potentiels et des contraintes liées à cette recherche.
- Je certifie être affilié(e) à un régime de sécurité sociale ou bénéficiaire d'un tel régime, sauf dérogation exceptionnelle.
- J'ai le droit de refuser de participer à la recherche ou de retirer mon consentement à tout moment sans conséquence sur ma prise en charge médicale et sans encourir aucune responsabilité ni préjudice de ce fait.
- J'ai bien compris la possibilité qui m'est réservée d'interrompre ma participation à cette recherche à tout moment sans avoir à justifier ma décision et j'informerai l'investigateur qui me suit dans la recherche. Cela ne remettra pas en cause la qualité des soins ultérieurs.
- J'ai bien compris que l'investigateur peut interrompre à tout moment ma participation à la recherche s'il le juge nécessaire.
- J'ai bien noté que je dispose d'un droit d'accès, de rectification, de limitation et, le cas échéant, d'opposition et d'effacement, concernant le traitement de mes données personnelles. Ces droits s'exercent en premier lieu auprès de l'investigateur qui me suit dans le cadre de cette recherche et qui connaît mon identité.
- J'ai bien pris connaissance que cette recherche est autorisée par l'Agence Nationale de Sécurité du Médicament et des produits de santé (ANSM) et a reçu l'avis favorable du Comité de Protection des Personnes. Le promoteur de la recherche a souscrit une assurance de responsabilité civile en cas de préjudice auprès de la société LLOYD'S INSURANCE COMPANY S.A .
- Mon consentement ne décharge en rien l'investigateur et le promoteur de la recherche de leurs responsabilités à mon égard. Je conserve tous mes droits garantis par la loi.
- Les résultats globaux de la recherche me seront communiqués à la fin de la recherche, si j'en fais la demande auprès de l'investigateur.
- Je ne pourrai pas participer à une autre recherche pendant une période de *6 mois suivant la fin de ma participation à l'étude.*
- En cas d'examen susceptible de déceler des anomalies, je consens à être tenu informé(e) des informations relatives à mon état de santé et des éventuelles anomalies qui pourraient être décelées à l'occasion de la recherche.

- Après le commencement de la recherche, je pourrais à tout moment demander des informations complémentaires au Dr Etienne BUSCAIL.
- Deux exemplaires originaux de ce formulaire de consentement ont été établis : un m'a été remis, le second gardé par l'investigateur. Ils seront conservés dans le dossier de l'étude au minimum 30 ans après la fin de la recherche.
- J'ai été informé(e) sur la façon dont mes données personnelles et mes échantillons biologiques pourront être collectés, utilisés et partagés comme décrit dans ce document.

En cas de nécessité pour la continuité de vos soins et votre suivi, j'accepte que mon médecin traitant soit informé de ma participation à cette recherche	Oui ☐ Non ☐
J'accepte que mes données personnelles codées soient utilisées pour d'autres recherches liées à la santé ou à la médecine (dans le domaine de la santé digestive), exclusivement à des fins scientifiques sachant que je peux à tout moment retirer mon accord.	Oui ☐ Non ☐

Signature du/de la participant(e) :	Date de signature :<br><br>|__|__| / |__|__| / |__|__|__|__|
Prénom et Nom du/de la participant(e) en majuscules :	Date de naissance du/de la participant(e) (pour éviter les homonymies)<br><br>|__|__| / |__|__| / |__|__|__|__|
Signature de l'investigateur ou du médecin / professionnel de santé qui le représente ayant informé le/la participant(e) :	Date de signature :<br><br>|__|__| / |__|__| / |__|__|__|__|
Prénom et nom de l'investigateur ou du médecin / professionnel de santé qui le représente ayant informé le/la participant(e) :	
