# Supplementary material for: Rationale and design of the AlloFIST trial: a phase I/IIa study to evaluate dose escalation of allogeneic adipose-derived stroma/stem cells for the treatment of Crohn’s fistula
Source: BMJ Open. 2025 Dec 29;15(12):e104517. doi: 10.1136/bmjopen-2025-104517 (PMC12750760; doi:10.1136/bmjopen-2025-104517)
Supplement: Supplementary file 3 [file bmjopen-2025-104517.reviewer_comments.pdf]

PEER REVIEW HISTORY

BMJ Open publishes all reviews undertaken for accepted manuscripts. Reviewers are asked to complete a checklist review form and are provided with free text boxes to elaborate on their assessment. These free text comments are reproduced below.

ARTICLE DETAILS

Title (Provisional)

Rationale and Design of the AlloFIST Trial: A phase I/IIa Study to Evaluate Dose Escalation of Allogeneic Adipose-Derived Stroma/Stem Cells for the Treatment of Crohn's Fistula

Authors

Buscail, Etienne; Gilletta de saint joseph, Cyrielle; Lebrin, Marine; Frument, Ines; Gross, fabian; Bournet, Barbara; Buscail, Louis; Culetto, Adrian; Mokrane, Fatima; Delchier, Marie Charlotte; Quelven, isabelle; Daguzan, Charline; Pugnet, Grégory; Duffas, Jean Pierre; Ghouti, Laurent; Philis, Antoine; Carrere, Nicolas; Lepage, benoit; Le Cosquer, guillaume

---

VERSION 1 - REVIEW

**Reviewer**	**1**
**Name**	**Gómez-Ferrer, Marta**
**Affiliation**	**Instituto de Investigación Sanitaria La Fe**
**Date**	**12-Aug-2025**
**COI**	**None**

Thank you for the opportunity to review this manuscript, which addresses an important and challenging clinical problem: the treatment of complex perianal fistulas in patients with Crohn's disease refractory to conventional therapies. The authors presented a phase I/IIa dose-escalation trial evaluating the safety and efficacy of adipose-derived mesenchymal stromal/stem cells (CellReady®) in this population. The topic is timely and of considerable interest, given the limited curative options currently available. However, there are several points that require clarification and further elaboration to improve the scientific rigor, transparency, and interpretability of the work.

Comments

Thank you for the opportunity to review this manuscript, which addresses an important and challenging clinical problem: the treatment of complex perianal fistulas in patients with Crohn's disease refractory to conventional therapies. The authors presented a phase I/IIa dose-escalation trial

evaluating the safety and efficacy of adipose-derived mesenchymal stromal/stem cells (CellReady®) in this population. The topic is timely and of considerable interest, given the limited curative options currently available. However, there are several points that require clarification and further elaboration to improve the scientific rigor, transparency, and interpretability of the work.

Minor questions and comments

- Across different sections, the values reported in scientific notation do not consistently match or are misspelled. Please check them.

L68 L93 ➔ (5.10^7 and 10.10^7 cells) replace with (5×10^7 and 10×10^7 cells)
L111 ➔ number of cells (1 to 12,107) replace with (1 to 12×10^7)
L164 ➔ AdMSC: 5,107 and 10,107 replace with (5×10^7 and 10×10^7 cells) L297 ➔
$12.5{*}10^6$ AdMSC/vial replace with 12.5×10^6 AdMSC/vial

- L94 Since what is described as original, it would be interesting to mention the maximum passage number of cells that is injected.

- L117-L118 As the authors correctly mention, a retrospective study of the Spanish clinical trial was carried out to extend the follow-up of these patients for more than 3 years "4. Guadalajara H, Herreros D, De-La-Quintana P, *et al.* Long-term follow-up of patients undergoing adipose-derived adult stem cell administration to treat complex perianal fistulas. *Int J Colorectal Dis*. 2012;27:595–600. doi: 10.1007/s00384-011-1350-1", which confirmed an excellent safety profile. I believe it is misleading to say that "Previous studies by Korean and Spanish teams have evaluated the feasibility, safety and efficacy of the cells **at the 2-month follow-up visit**.

-L288-L290 Data are lacking that would allow for reproducibility of the experiments if the results obtained in the study are to be replicated. This is very important to avoid future errors by other centers in more advanced phases. How are vials thawed, in a bath or at room temperature? In what vehicle (phosphate buffer saline, culture medium…) are they stored and at what temperature until injection? They mention "The transport conditions and quality of the product are checked" I assume the transport conditions refer to a suitable and constant temperature, but you don't mention it. What quality controls are performed on the cells before injection?

-L297-L300 The dosage of the injections is not clear, please define it better. I understand that for 50 million cells a 10 syringe is filled and for 100 million cells two 10 syringes. However, it is then said that "The content of two of the syringes is injected" in the case of the 50 million cells, is it just one syringe? Then it is mentioned that "the remaining half is injected" They only mention two syringes, what is the other half? Please, mention the vehicle in which the cells are dissolved.

Reviewer **2**

Name **Qiao, Yuqi**

Affiliation **Shanghai Jiao Tong University School of Medicine Affiliated Renji Hospital**

Date **03-Oct-2025**

COI **None**

This study demonstrates clear innovation and scientific validity in the field of allogeneic mesenchymal stem cell (MSC) therapy for Crohn's fistula, with particular strengths in the

refinement of cell products, BOIN (Bayesian Optimal Interval) dose design, and composite endpoint assessment design.

Key Limitations and Recommendations

1. Short follow-up duration: While 6 months meets the "minimum threshold" for Phase I safety, the peak of fistula recurrence occurs after 12 months. It is recommended to extend the follow-up period to 12 months as an exploratory endpoint for Phase IIa, so as to provide data on recurrence rates for Phase IIb.

2. Limitations in tumorigenicity monitoring: As a critical component of safety monitoring, in addition to extending the follow-up to 12 months, it is recommended to implement imaging re-evaluations at ≥ 12 months and classify "newly developed masses at the fistula site" as a separate serious adverse event (SAE).

3. Lack of immunogenicity monitoring: It is recommended to test anti-HLA-I/II antibodies and T cell proliferation responses in at least 3 subjects, to provide a basis for immunological safety in Phase II studies.

4. Unspecified in vivo cell survival/distribution: It is recommended to incorporate MRI tracking (e.g., with SPIO [Superparamagnetic Iron Oxide] labeling) or local tissue biopsy in 1–2 cases, to provide evidence for subsequent dose optimization.

5. Absence of early futility stopping rule: It is recommended to add an "efficacy futility" rule in Phase IIa (e.g., stop dose escalation if none of the first 3 subjects achieve partial response [PR]).

Other Minor Issues

1. In the flow diagram: The phrase "2 ou 3 patients with grade ≥ 2 adverse events (AEs) attributable to the treatment" should be corrected to "2 or 3".

From the perspective of Phase I/IIa studies, this research meets the requirements for safety exploration, dose escalation design, and efficacy signal capture. However, there remains room and a need for improvement in certain aspects, and the above suggestions are for reference only.

VERSION 1 - AUTHOR RESPONSE

Reviewer 1 Dr. Marta Gómez-Ferrer, Instituto de Investigación Sanitaria La Fe

Comments

Thank you for the opportunity to review this manuscript, which addresses an important and challenging clinical problem: the treatment of complex perianal fistulas in patients with Crohn's disease refractory to conventional therapies. The authors presented a phase I/IIa dose-escalation trial evaluating the safety and efficacy of adipose-derived mesenchymal stromal/stem cells (CellReady®) in this population. The topic is timely and of considerable interest, given the limited curative options currently available.

However, there are several points that require clarification and further elaboration to improve the scientific rigor, transparency, and interpretability of the work.

RE: We would like to thank the reviewer for the positive evaluation of our work.

Minor questions and comments

- Across different sections, the values reported in scientific notation do not consistently match or are misspelled. Please check them.
L68 L93→(5.107 and 10.107 cells) replace with (5 × 107 and 10 × 107 cells) L111 → number of cells (1 to 12,107) replace with (1 to 12 × 107) L164→AdMSC: 5,107 and 10,107 replace with (5 × 107 and 10 × 107 cells) L297 → 12.5*106 AdMSC/vial replace with 12.5 × 106 AdMSC/vial

RE: Done

- L94 Since what is described as original, it would be interesting to mention the maximum passage number of cells that is injected.

RE: Passage 1 is the working cell bank, then Cellready culture passages 2 and 3 before the Cellready drug substance.

- L117-L118 As the authors correctly mention, a retrospective study of the Spanish clinical trial was carried out to extend the follow-up of these patients for more than 3 years "4. Guadalajara H, Herreros D, De-La-Quintana P, et al. Long-term follow-up of patients undergoing adipose- derived adult stem cell administration to treat complex perianal fistulas. Int J Colorectal Dis. 2012;27:595–600. doi: 10.1007/s00384-011-1350-1", which confirmed an excellent safety profile. I believe it is misleading to say that "Previous studies by Korean and Spanish teams have evaluated the feasibility, safety and efficacy of the cells at the 2-month follow-up visit.

RE: We agree and we have corrected in the text.

-L288-L290 Data are lacking that would allow for reproducibility of the experiments if the results obtained in the study are to be replicated. This is very important to avoid future errors by other centers in more advanced phases. How are vials thawed, in a bath or at room temperature?
In what vehicle (phosphate buffer saline, culture medium...) are they stored and at what temperature until injection? They mention "The transport conditions and quality of the product are checked" I assume the transport conditions refer to a suitable and constant temperature, but you don't mention it. What quality controls are performed on the cells before injection?

RE: We agree and corrected the main text as follow:" The doses will be stored in nitrogen gas at Cell-Easy (-150°C). A few days before a patient's injection, the frozen cell doses will be shipped to the Toulouse hospital pharmacy specialized in Advanced therapy medicinal products management in a special dry-shipper container containing liquid nitrogen with continuous temperature monitoring. The thawed cells will be stored at 2-8°C until they are dispensed to the investigator team for injection within a

maximum of 10 hours, in accordance with the stability studies conducted on the reconstituted product in the syringe. Transport conditions will comply with Good Transport Practices (in accordance with European Directive 2004/23/EC). Frozen cell doses will be stored at Cell-Easy and then sent to the Toulouse hospital pharmacy specialized in Advanced therapy medicinal products on the day of a patient's injection. The thawed product in the syringe will be checked by a pharmacist and then dispensed by authorized personnel. All checks are carried out by the manufacturer before shipment and then by the hospital pharmacist upon receipt and before dispensing."

-L297-L300 The dosage of the injections is not clear, please define it better. I understand that for 50 million cells a 10 syringe is filled and for 100 million cells two 10 syringes. However, it is then said that "The content of two of the syringes is injected" in the case of the 50 million cells, is it just one syringe? Then it is mentioned that "the remaining half is injected" They only mention two syringes, what is the other half? Please, mention the vehicle in which the cells are dissolved.

RE: We agree and corrected the main text as follow:" For 50 million cells, there are two 10 ml syringes, and for 100 million cells, there are four 10 ml syringes. The final volume of the cell suspension is 2.5 mL (overfilled to 2.7 mL) and the medium consists of DMEM containing human albumin and Cryostor® CS 10."

Reviewer: 2
Dr. Yuqi Qiao, Shanghai Jiao Tong University School of Medicine Affiliated Renji Hospital
Comments to the Author:
This study demonstrates clear innovation and scientific validity in the field of allogeneic mesenchymal stem cell (MSC) therapy for Crohn's fistula, with particular strengths in the refinement of cell products, BOIN (Bayesian Optimal Interval) dose design, and composite endpoint assessment design.

RE: We would like to thank the reviewer for the positive evaluation of our work.

Key Limitations and Recommendations

1. Short follow-up duration: While 6 months meets the "minimum threshold" for Phase I safety, the peak of fistula recurrence occurs after 12 months. It is recommended to extend the follow-up period to 12 months as an exploratory endpoint for Phase IIa, so as to provide data on recurrence rates for Phase IIb.

2. Limitations in tumorigenicity monitoring: As a critical component of safety monitoring, in addition to extending the follow-up to 12 months, it is recommended to implement imaging re-evaluations at ≥ 12 months and classify "newly developed masses at the fistula site" as a separate serious adverse event (SAE).

3. Lack of immunogenicity monitoring: It is recommended to test anti-HLA-I/II antibodies and T cell proliferation responses in at least 3 subjects, to provide a basis for immunological safety in Phase II studies.

4. Unspecified in vivo cell survival/distribution: It is recommended to incorporate MRI tracking (e.g., with SPIO [Superparamagnetic Iron Oxide] labeling) or local tissue biopsy in 1–2 cases, to provide evidence for subsequent dose optimization.

5. Absence of early futility stopping rule: It is recommended to add an "efficacy futility" rule in Phase IIa (e.g., stop dose escalation if none of the first 3 subjects achieve partial response [PR]).

Response to the five comments:

Thank you for your comment. As the research protocol has already been registered with the relevant French hospital and public health authorities, we are unable to modify it. Nevertheless, we would like to thank the reviewer for their comments and observations.

Other Minor Issues
1. In the flow diagram: The phrase "2 ou 3 patients with grade ≥ 2 adverse events (AEs) attributable to the treatment" should be corrected to "2 or 3".

RE: Done

From the perspective of Phase I/IIa studies, this research meets the requirements for safety exploration, dose escalation design, and efficacy signal capture. However, there remains room and a need for improvement in certain aspects, and the above suggestions are for reference only.

VERSION 2 - REVIEW

Reviewer 2

Name Qiao, Yuqi

Affiliation Shanghai Jiao Tong University School of Medicine Affiliated Renji Hospital

Date 22-Nov-2025

COI

There are no serious design issues with the research protocol in the manuscript, and it is suitable for publication.
